# COMPARISON OF DNA METHYLATION IN SALIVA, BUFFY COAT AND PBMC SAMPLES IN CATSLIFE

**DOI:** 10.1093/geroni/igad104.3240

**Published:** 2023-12-21

**Authors:** Donald Evans, Andrew Smolen, Chandra Reynolds

**Affiliations:** University of Colorado Boulder, Boulder, Colorado, United States; University of Colorado Boulder, Boulder, Colorado, United States; University of Colorado Boulder, Boulder, Colorado, United States

## Abstract

The design of the Colorado Adoption/Twin Study of Lifespan behavioral development and cognitive aging (CATSLife) allows for separation of genetic and environmental factors affecting health and cognitive aging. Currently, this sample is transitioning through midlife. One goal of this study is to identify epigenetic changes associated with aging, however methylation profiles differ across tissue types. Therefore, we describe here the analysis of DNA methylation in three CATSLife sample types: saliva, buffy coat, and peripheral blood mononuclear cells (PBMCs). Obtaining saliva is least invasive, although it may be less informative about brain and cognitive aging processes. In contrast, while buffy coats and PBMCs may be more informative, obtaining blood is more invasive. Buffy coats are less expensive to obtain than PBMCs, however they contain more cell types which may complicate analysis. Our analyses of 91 participants, suggested that saliva samples were less likely to pass initial quality control (< 96% CpGs detected). Unsurprisingly, the buffy coat and PBMC DNA methylation signatures are more similar to each other (r=0.97) than either is to saliva (rs=0.88—0.92). In initial analyses, each sample type demonstrated expected familial/heritable patterns of similarity across sites (MZ twins > DZ twins > biological & adoptive siblings). Given the additional cost of isolating PBMCs and computational methods to account for complex cell mixtures, the use of buffy coat DNA increases accessibility to analyze epigenetic changes longitudinally, enabling the early identification of epigenetic biomarkers of cognitive decline and the effect of both genes and environments on this process.

